# Chemical Basis of Metabolic Network Organization

**DOI:** 10.1371/journal.pcbi.1002214

**Published:** 2011-10-13

**Authors:** Qiang Zhu, Tao Qin, Ying-Ying Jiang, Cong Ji, De-Xin Kong, Bin-Guang Ma, Hong-Yu Zhang

**Affiliations:** 1 National Key Laboratory of Crop Genetic Improvement, College of Life Science and Technology, Huazhong Agricultural University, Wuhan, China; 2 Center for Bioinformatics, Huazhong Agricultural University, Wuhan, China; 3 Shandong Provincial Research Center for Bioinformatic Engineering and Technique, School of Life Sciences, Shandong University of Technology, Zibo, China; University of Virginia, United States of America

## Abstract

Although the metabolic networks of the three domains of life consist of different constituents and metabolic pathways, they exhibit the same scale-free organization. This phenomenon has been hypothetically explained by preferential attachment principle that the new-recruited metabolites attach preferentially to those that are already well connected. However, since metabolites are usually small molecules and metabolic processes are basically chemical reactions, we speculate that the metabolic network organization may have a chemical basis. In this paper, chemoinformatic analyses on metabolic networks of Kyoto Encyclopedia of Genes and Genomes (KEGG), *Escherichia coli* and *Saccharomyces cerevisiae* were performed. It was found that there exist qualitative and quantitative correlations between network topology and chemical properties of metabolites. The metabolites with larger degrees of connectivity (hubs) are of relatively stronger polarity. This suggests that metabolic networks are chemically organized to a certain extent, which was further elucidated in terms of high concentrations required by metabolic hubs to drive a variety of reactions. This finding not only provides a chemical explanation to the preferential attachment principle for metabolic network expansion, but also has important implications for metabolic network design and metabolite concentration prediction.

## Author Summary

The metabolic networks of the three domains of life exhibit the same scale-free organization, which has been hypothetically explained in terms of preferential attachment principle. Here we reveal that the scale-free organization of metabolic networks may have a chemical basis. Through a chemoinformatic analysis on metabolic networks of Kyoto Encyclopedia of Genes and Genomes (KEGG), *Escherichia coli* and *Saccharomyces cerevisiae*, it was found that the metabolites with higher degrees of connectivity (hubs) are of relatively stronger polarity. The reason underlying this phenomenon is that to drive a variety of reactions, metabolic hubs have to be highly concentrated. Since the intracellular environments are hydrophilic, metabolic hubs have to be strong-polar to reach high concentrations. This finding has direct implications for metabolic network design and provides a chemical explanation to the preferential attachment principle, which has been validated by numerical simulations of metabolic network expansion. In addition, the correlations between metabolite concentration, metabolic network topology and metabolite chemical properties also suggest that we can use chemical and topological properties of metabolites to predict their intracellular concentrations. A support vector regression model has been successfully established to predict the metabolite concentrations for *Escherichia coli*.

## Introduction

One of the most intriguing findings in systems biology is that despite the varied constituents and metabolic pathways of three domains of life, their metabolic networks exhibit the same scale-free organization. That is, a small part of metabolites participate in a large number of reactions (which are also termed hubs), while others are involved in a few reactions [1]. As the scale-free architectures are robust and error-tolerant, this finding provides meaningful insights into the design principle of metabolic networks.

The scale-free organization of metabolic networks has been hypothetically explained in terms of evolution that the new-recruited metabolite members attach preferentially to those that are already well connected (rich get richer, also known as preferential attachment principle) [2]–[4]. This implies that the metabolic network hubs originated relatively earlier than others in evolutionary history [5]. However, several issues about this evolutionary explanation remain elusive. First, the molecular basis of preferential attachment principle has not been fully elucidated, as it is inexplicable how the new metabolites “know” which metabolites are well connected. Second, the evolutionary explanation to the metabolic network organization has little implications for network design, because we do not know how to choose metabolites as hubs to construct a new metabolic network. Since most metabolites are small molecules and metabolic processes are basically chemical reactions, we speculate that the metabolic network organization may have a chemical basis, which stimulated our interest to address these issues by combining bioinformatics and chemoinformatics. The latter is a discipline devoted to encoding, storing, managing, searching and analyzing all kinds of chemical data by information technology [6], [7].

## Results/Discussion

### Correlations between network topology and chemical properties

**Figure 1:**
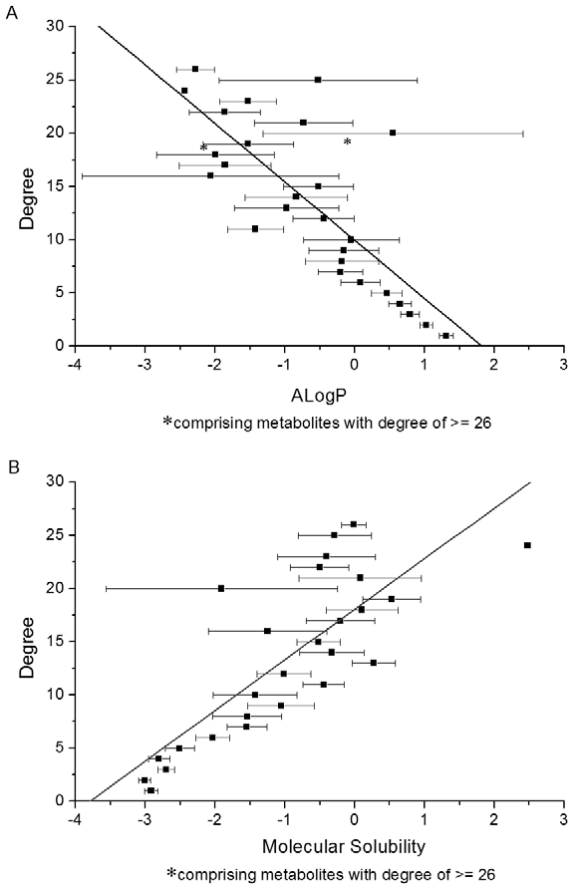
Correlations between topological and chemical properties of KEGG metabolites. (**A**) Degree-ALogP (mean ± SE) correlation for KEGG metabolites (*R* = −0.778, *P*<0.001). (**B**) Degree-Molecular Solubility (mean ± SE) correlation for KEGG metabolites (*R* = 0.795, *P*<0.001).

**Figure 2:**
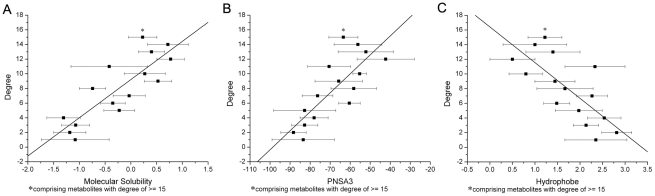
Correlations between topological and chemical properties of *E. coli* metabolites. (**A**) Degree-Molecular Solubility (mean ± SE) correlation (*R* = 0.835, P<0.001). (**B**) Degree-PNSA3 (mean ± SE) correlation (*R* = 0.796, *P*<0.001). (**C**) Degree-Hydrophobe (mean ± SE) correlation (*R* = −0.743, *P*<0.005). PNSA3 is defined as atomic charge weighted partial negative surface area. Hydrophobe is the number of hydrophobe.

Primarily, we explored the relationships between network topology and chemical properties for the metabolites recorded in Kyoto Encyclopedia of Genes and Genomes (KEGG). As illustrated in Figure S1, the metabolic network of KEGG is scale-free. There are 154 metabolites with degrees (defined as the number of edges linked to the metabolites) higher than 10, while 1180 are connected with only one metabolite. As shown in Table 1 and Figure 1, there exist qualitative and even quantitative correlations between degree and some chemical properties. In particular, molecular polarity, characterized by partition coefficients (ClogP, AlogP and LogD), ratio of atomic charge weighted partial positive surface area on total molecular surface area (FPSA3) and water solubility, rises with the increase of degree. Similar correlations can be observed for the metabolic networks of *Escherichia coli* (*E. coli*) (Figure 2) and *Saccharomyces cerevisiae* (*S. cerevisiae*) (Table 2). Therefore, it seems that metabolites get more polar and thus more water-soluble with the rise of degrees, which implies that the organization of the metabolic networks has a chemical basis. It is of apparent interest to explore the reasons underlying these correlations.

**Table 1:** Mean values of some chemical descriptors for KEGG-recorded metabolites.

Descriptors	Characterization	Mean values		
		Degree 1 (n = 1180)	Degree 2-6 (n = 3327)	Degree > 6 (n = 368)
ClogP^a^	Partition coefficient octanol/water	1.30^d^	0.70^d^	−1.10^d^
FPSA3^b^	Ratio of atomic charge weighted partial positive surface area on total molecular surface area	0.062^d^	0.067^d^	0.079^d^
LogD^c^	Octanol-water partition coefficient calculated taking into account the ionization states of the molecule	0.43^d^	−0.53^d^	−2.31^d^
Molecular Solubility^c^	Water solubility, expressed as logS, where S is the solubility in mol/L	−2.91^d^	−2.82^d^	−0.98^d^

a calculated with Cerius2 (Version 4.11L. Accelrys Inc. San Diego, CA.).

b calculated with Sybyl (Version 7.0. Tripos Associates Inc. St. Louis, MO.).

c calculated with Pipeline Pilot (Student Edition. Version 6.1.5. SciTegic Accelrys Inc. San Diego, CA.).

d Kruskal-Wallis Test significance at the 0.01 level.

**Table 2:** Mean values of some chemical descriptors for *S. cerevisiae* metabolites.

Descriptors	Characterization	Mean values		
		Degree 1-3 (n = 301)	Degree 4-15 (n = 285)	Degree > 15 (n = 26)
ClogP^a^	Partition coefficient octanol/water	0.46^d^	−0.54^d^	−3.05^d^
FPSA3^b^	Ratio of atomic charge weighted partial positive surface area on total molecular surface area	0.066^d^	0.068^d^	0.080^d^
LogD^c^	Octanol-water partition coefficient calculated taking into account the ionization states of the molecule	−0.89^e^	−1.94^e^	−3.88^e^
Molecular Solubility^c^	Water solubility, expressed as logS, where S is the solubility in mol/L	−2.47^e^	−1.99^e^	0.11^e^

a calculated with Cerius2 (Version 4.11L. Accelrys Inc. San Diego, CA.

b calculated with Sybyl (Version 7.0. Tripos Associates Inc. St. Louis, MO.).

c calculated with Pipeline Pilot (Student Edition. Version 6.1.5. SciTegic Accelrys Inc. San Diego, CA.).

d Kruskal-Wallis Test significance at the 0.05 level.

e Kruskal-Wallis Test significance at the 0.01 level.

### Explanation to the correlations between network topology and chemical properties

As metabolic reactions are basically chemical reactions, it is natural to resort to chemical principles to explain the correlations. It is well known that the precondition for a chemical reaction to occur is Δ*G*  =  Δ*G* ^0^ *+ RT*ln*Q* <0, where *Q* is the reaction quotient and is determined by the relative concentrations of reactants and products. Thus, for metabolites that participate in a large number of reactions as reactants (which usually have large degrees, as shown in Table S4), they must reserve high concentrations (quantities) to drive the reactions. Since metabolic reactions mainly occur in non-membrane systems which are hydrophilic environments, the metabolic network hubs must be highly water-soluble to reach high concentrations, which means that the hubs tend to be strong-polar. Therefore, the observed correlations between degree and chemical properties could be basically explained in terms of chemical property requirements of metabolic hubs. This explanation is supported by the correlations between degree and metabolite concentration and between metabolite concentration and chemical properties.

**Figure 3:**
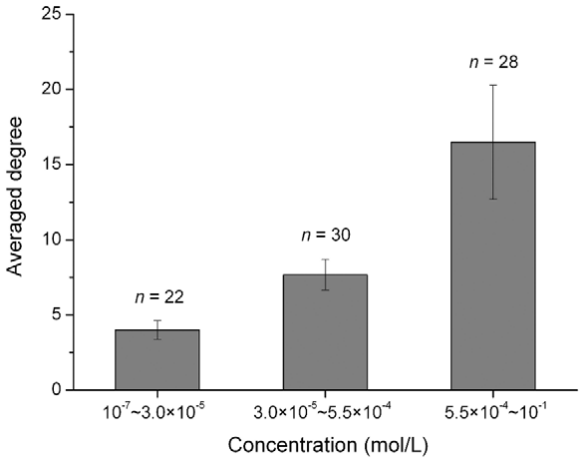
Degree-concentration correlation for *E. coli* metabolites (*P*<0.01, Kruskal-Wallis test).

Recently, the absolute concentrations for over 100 metabolites of *E. coli*, exponentially growing in aerobic environment, were determined by Bennett and co-workers [8]. The concentrations of the measured metabolites are strongly biased. The top 10 abundant compounds account for 77% of the total concentration, while the less abundant half comprise only 1.3%, reminiscent of the topological structures of metabolic networks. As shown in Figure 3, there exists a correlation between the concentration and degree for *E. coli* metabolites. The metabolites with larger degrees have relatively higher concentrations and the degrees decline gradually with the drop of concentrations. However, one may argue that the metabolite concentrations oscillate during different phases of life, so how the concentrations of metabolites can correlate with degrees of connectivity–a static property? The answer resides in the fact that the amplitude of metabolite oscillation is rather low. For instance, during the life cycle of a yeast cell the amplitude of metabolite oscillation is usually within 10-fold, with a median of ∼2.4-fold [9]. Therefore, it is reasonable to consider that the observed correlation between degree and metabolite concentration (at the level of order of magnitude) is robust.

**Figure 4:**
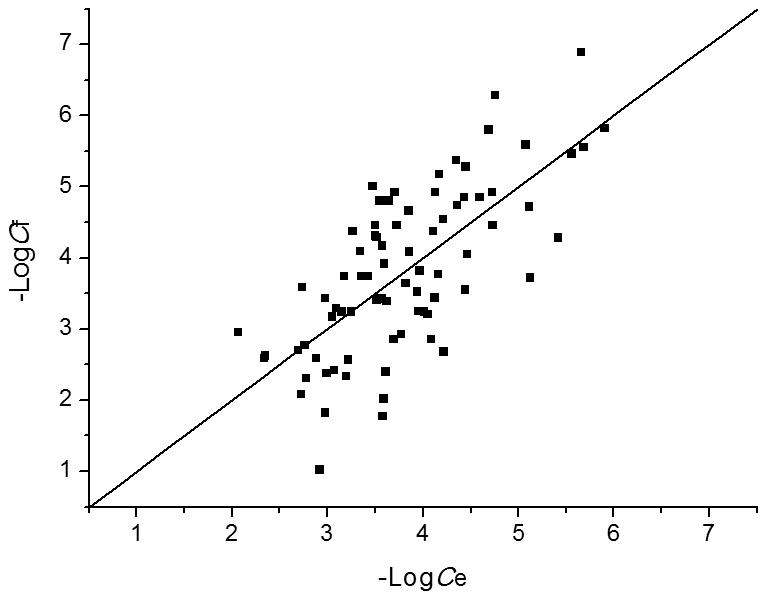
Theoretical fitting of *E. coli* metabolite concentrations by chemical properties. A stepwise multiple linear regression analysis was conducted to select the most meaningful chemical properties that correlate with concentration (*C*). The final regression equation is: −Log*C* = 6.105 + 0.431 × "ClogP" + 15.595 × "FNSA3" + 16.727 × "FPSA3" − 5.333 × "RPCG". The negative logarithm of fitted concentrations (−Log*C*_f_) for 80 *E. coli* metabolites correlates well with that of experimental values (−Log*C*_e_) (*R* = 0.704, *P*<0.0001).

A stepwise multiple linear regression analysis was conducted by SPSS (Version 15.0. SPSS Inc. Chicago, IL.) to select the most meaningful chemical properties from 83 descriptors to correlate with negative logarithm of *E. coli* metabolite concentrations (−Log*C*). The final regression equation is: −Log*C*  =  6.105 + 0.431 × "ClogP" + 15.595 × "FNSA3" + 16.727 × "FPSA3" − 5.333 × "RPCG", in which ClogP, FNSA3 (ratio of atomic charge weighted partial negative surface area on total molecular surface area), FPSA3 and RPCG (ratio of most positive charge on sum total positive charge) are all descriptors characterizing molecular polarity. The fitted concentrations by the chemical properties correlate well with the experimental values (Figure 4), indicating that the metabolite concentrations (at least for *E. coli*) are determined to a certain extent by their polarity and solubility, namely, strong-polar metabolites have relatively high concentrations. This finding is similar to the observation about protein abundance of *E. coli* that highly abundant proteins are on average more hydrophilic than those with low copy numbers [10]. However, in protein-protein interaction (PPI) networks, protein degree is negatively correlated with concentration [11], just contrary to the observation on metabolic networks. The underlying reason was suggested as that the hub proteins of PPI networks tend to use hydrophobic residues at surface to bind diverse partners through nonspecific hydrophobic interactions [11]. The cellular concentrations of hub proteins are thus constrained by their hydrophobicity. Therefore, the different behaviors of PPI and metabolic network hubs can be well understood by basic chemical rules.

Taken together, the above observations offer an explanation to the correlation between topology and chemistry of metabolic networks. This finding also provides new clues to understanding the molecular basis of preferential attachment principle underlying the evolution of metabolic networks.

### Chemical basis for the preferential attachment principle

Since life originated from water environments, the primordial metabolites must be highly hydrophilic. With the evolution of organisms, more and more complex membrane systems evolved, which required hydrophobic metabolites to perform intercellular and intracellular communications [12]. As a result, the evolutionary direction of metabolites is from hydrophilic to hydrophobic, which is clearly shown in the chemical evolution of *S. cerevisiae* metabolomes (Table 3). According to the correlation between metabolite concentration and chemical properties (Figure 4), it is reasonable to infer that the early-originated metabolites have relatively higher concentrations than the late-recruited counterparts in water environments. Since high-concentrated metabolites have more potential to drive new reactions, it is understandable why the new-recruited metabolites prefer to select old members as initial reactants (because they are more abundant and thus more accessible). Taken together, the present analysis reveals that metabolite concentration is a key factor to govern the metabolic network expansion. Although the late metabolites can not “know” which counterpart is well connected, they can “sense” which member is abundant, which provides a self-consistent explanation to the preferential attachment principle in terms of chemistry.

**Table 3:** Mean values of some chemical descriptors for early and late metabolites of *S. cerevisiae*.

Descriptors	Characterization	Mean values	
		Early metabolites (n = 243)	Late metabolites (n = 369)
ClogP^a^	Partition coefficient octanol/water	−1.98^d^	0.98^d^
FPSA3^b^	Ratio of atomic charge weighted partial positive surface area on total molecular surface area	0.079^d^	0.061^d^
LogD^c^	Octanol-water partition coefficient calculated taking into account the ionization states of the molecule	−3.12^d^	−0.44^d^
Molecular Solubility^c^	Water solubility, expressed as logS, where S is the solubility in mol/L	−0.74^d^	−3.06^d^

a calculated with Cerius2 (Version 4.11L. Accelrys Inc. San Diego, CA.).

b calculated with Sybyl (Version 7.0. Tripos Associates Inc. St. Louis, MO.).

c calculated with Pipeline Pilot (Student Edition. Version 6.1.5. SciTegic Accelrys Inc. San Diego, CA.).

d Mann-Whitney Test significance at the 0.01 level.

**Figure 5:**
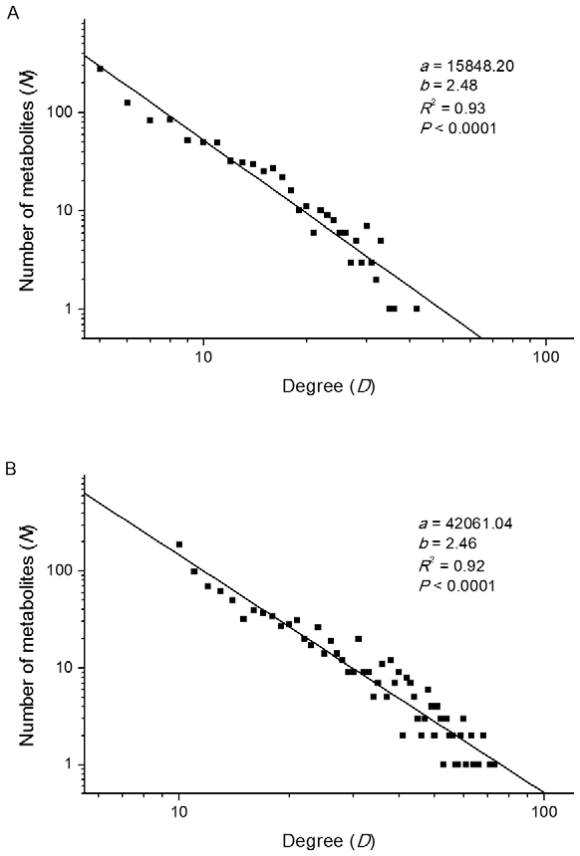
Numerical simulations of metabolic network expansion. The simulations were based on three rules: i) *n* metabolites are added in each expansion step (*n* = 1 in the present simulations); ii) the newly added metabolites have lower concentrations compared to the old ones; iii) the metabolites of higher concentrations have higher probability to be involved in the emerging reactions (edges). The simulations start with 1 metabolite with the initial concentration (*C*_i_) of 1,000,000 and terminate when a metabolite reaches a concentration (*C*_f_) of ≤ 10. The concentration decline (*d*) in each step is 1,000, with a random fluctuation (*f*) of 1,500. (**A**) The number of reactions (edges) added in each step is 5; (**B**) The number of reactions (edges) added in each step is 10. In both simulations, the number of metabolites (*N*) decays with the increase of degrees (*D*) and follows the equation *N* = *aD^-b^*.

This explanation was validated by numerical simulations that were based on three rules. First, the network expands continuously by adding new metabolites (vertices) with a constant rate, namely, *n* metabolites are added in each step (*n*  =  1 in the present simulations). Second, the newly added metabolites have lower concentrations compared to the old ones, *i.e.*, there is a declining trend for the concentrations of emerging metabolites. Third, the metabolites of higher concentrations have higher probability to be involved in the emerging reactions (edges). The present simulations start with 1 metabolite with the initial concentration (*C*_i_) of 1,000,000 and terminate when a metabolite reaches a concentration (*C*_f_) of ≤ 10. This concentration range spans five orders of magnitude, which coincides with the variation range of metabolite concentrations in *E. coli* (from ∼10^−7^ to ∼10^−2^ mol/L) [8]. The concentration decline (*d*) in each step is 1,000, with a random fluctuation (*f*) of 1,500. As a result, the total number of generated metabolites reaches around 1,000, which is close to the real number of metabolites of organisms. The numbers of reactions (edges) added in each step are 5 or 10. As shown in Figure 5, the simulations with different parameters exhibit similar power-law distributions of node degrees, which suggests that the concentration-governed model provides a viable explanation to the scale-free organization of metabolic networks.

### Implications for metabolic network design

The above finding implies a chemical criterion in metabolic network design that the polarity of hubs should be compatible with the working environments to guarantee the high concentrations of these critical metabolites. If the environments are polar (*e.g.*, water), one should use hydrophilic molecules as hubs, while if the environments are non-polar (*e.g.*, hydrocarbon solutions) [13], hydrophobic molecules should be selected as hubs. This opinion is preliminarily supported by the fact that the “core” of organic chemical network (*i.e.*, a small set of strongly connected, chemically diverse substances) identified by Bishop *et al.* [14] are really much less polar than the hubs of metabolic networks (Table 4), well reflecting the fact that organic chemical reactions are mainly performed in organic solvents which are less polar than water. Thus, this chemical criterion is of apparent value in metabolic network design.

**Table 4:** Mean values of some chemical descriptors for hubs of KEGG-based network and cores of organic chemical network.

Descriptors	Characterization	Mean values	
		KEGG hubs (n = 279)	Chemical cores (n = 300)
ClogP^a^	Partition coefficient octanol/water	−1.26^d^	2.11^d^
FNSA3^b^	Ratio of atomic charge weighted partial negative surface area on total molecular surface area	−0.110^d^	−0.060^d^
FPSA3^b^	Ratio of atomic charge weighted partial positive surface area on total molecular surface area	0.080^d^	0.040^d^
LogD^c^	Octanol-water partition coefficient calculated taking into account the ionization states of the molecule	−2.56^d^	2.08^d^
Molecular Solubility^c^	Water solubility, expressed as logS, where S is the solubility in mol/L	−0.80^d^	−2.61^d^
RPCG^b^	Ratio of most positive charge on sum total positive charge (Relative positive charge)	0.158^d^	0.233^d^

a calculated with Cerius2 (Version 4.11L. Accelrys Inc. San Diego, CA.).

b calculated with Sybyl (Version 7.0. Tripos Associates Inc. St. Louis, MO.).

c calculated with Pipeline Pilot (Student Edition. Version 6.1.5. SciTegic Accelrys Inc. San Diego, CA.).

d Mann-Whitney Test significance at the 0.01 level.

### Implications for metabolite concentration prediction

A primary goal of systems biology is to quantitatively characterize cellular behaviors, which requires the information about the absolute concentrations of metabolites. As the intracellular content of metabolites is quite low [15], it is a big challenge to determine their concentrations experimentally. Thus, it is of great significance to use theoretical methods to do predictions. In a pioneering study, Kümmel *et al* established a network-embedded thermodynamic (NET) method to predict intracellular metabolite concentrations [16]. However, this method depends largely on Gibbs energies of formation for metabolites, so its use is restricted to a small part of metabolites. The correlations between metabolite concentration and their topological/chemical properties revealed in this study suggest that intracellular metabolite concentrations may be predicted by their topological and chemical properties.

**Figure 6:**
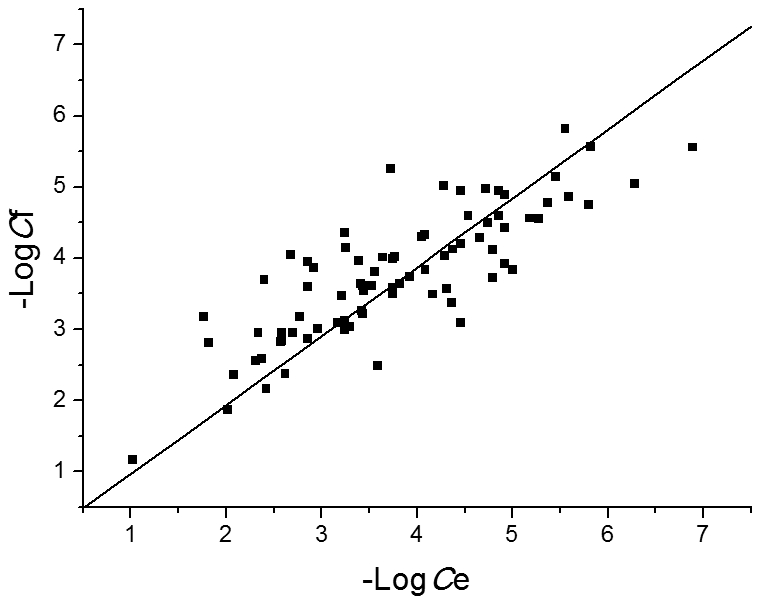
Theoretical fitting of *E. coli* metabolite concentrations by the SVR model. The negative logarithm of fitted concentrations (−Log*C*_f_) for 80 *E. coli* metabolites correlates well with that of experimental values (−Log*C*_e_): −Log*C*_f_ = 0.9678 × −Log*C*_e_ (*R* = 0.827, *P*<0.0001, regression without intercept).

By using the support vector regression (SVR) [17] method in R (version 2.11.1), a SVR model was established to predict *E. coli* metabolite concentrations by their topological and chemical properties. This model was evaluated by leave-one-out cross validation. The squared correlation coefficient is 0.5906 and the total mean squared error is 0.5316. The fitted metabolite concentrations by this model correlate well with the original experimental values (Figure 6). To evaluate the relative contribution of each descriptor to the performance of SVR model, we constructed SVR models by deleting one parameter each time and calculated the squared correlation coefficients of leave-one-out cross validation by using grid search over supplied parameter ranges. The smaller the squared correlation coefficient becomes, the more important the deleted descriptor is to the SVR model. As shown in Table 5, the deletion of degree results in the lowest squared correlation coefficient, followed by the deletion of ClogP, which means that degree and ClogP make most important contributions to the performance of SVR model.

**Table 5:** Performance of SVR models evaluated by descriptor deletion.

Deleted descriptor	Characterization	Squared correlation coefficient^f^	Total mean squared error^f^
Degree^a^	Number of edges linked to the node of network	0.4547	0.7094
ClogP^b^	Partition coefficient octanol/water	0.5185	0.6304
Amide Molecules^c^	Number of amide	0.5489	0.5952
N Count^c^	Number of Nitrogen atoms	0.5674	0.5963
6mem rings Molecules^c^	Number of 6 membered rings	0.5680	0.5628
FNSA3^d^	Ratio of atomic charge weighted partial negative surface area on total molecular surface area	0.5691	0.5594
HBD Count^e^	Number of hydrogen bond donating groups in the molecule	0.5717	0.5744
FPSA3^d^	Ratio of atomic charge weighted partial positive surface area on total molecular surface area	0.5778	0.5482
ALogP^c^	The Ghose and Crippen octanol-water partition coefficient	0.5806	0.5449
LScore Molecules^c^	Floating point Lipinski measure	0.5860	0.5373
RPCG^d^	Ratio of most positive charge on sum total positive charge (Relative positive charge)	0.6045	0.5134

a calculated by Network Analyzer Plugin in Cytoscape-2.7.0.

b calculated with Cerius2 (Version 4.11L. Accelrys Inc. San Diego, CA.).

c calculated with Tripos Benchware DataMiner (Version 1.6. Tripos Associates Inc. St. Louis, MO.).

d calculated with Sybyl (Version 7.0. Tripos Associates Inc. St. Louis, MO.).

e calculated with Pipeline Pilot (Student Edition. Version 6.1.5. SciTegic Accelrys Inc. San Diego, CA.).

f derived from leave-one-out cross validation.

The *E. coli* metabolite concentrations that have been predicted by the NET method [16] were also estimated by the SVR model. The SVR predictions agree well with the NET results and those determined by prior experiments (at the level of order of magnitude) (Table 6). By the SVR method, the intracellular concentrations for other *E. coli* metabolites were also predicted and presented in Table S6, which can be used as initial data in *E. coli* metabolic network simulation. As the SVR model only depends on very basic (topological and chemical) properties of metabolites, it is expected to be applicable in metabolite concentration prediction for other bacteria.

**Table 6:** Comparison of predicted and experimental concentrations for some *E. coli* metabolites.

Metabolite^a^	Predicted concentration^b^	Predicted concentration^c^		Experimental concentration^d^
		Lower limit	Upper limit	
13DPG	n.a.^e^	3.237	3.959	n.d.^j^
2PG	3.347	3.292	3.770	2.394
3PG	3.260	2.387	2.495	2.394
3PHP	2.906	5.046	7.000	n.d.^j^
DHAP	3.221	3.155	3.252	3.174
F6P	3.416	3.796	6.000	3.319
G1P	3.935^f^	3.959	6.000	n.d.^j^
G6P	3.577^g^	3.301	3.523	3.319
G3P	3.170	4.301	5.046	3.174
R5P	3.341	3.959	4.699	3.824
RU5P	3.617^h^	3.824	4.699	3.824
X5P	3.594^i^	3.959	6.000	3.824

a Abbreviations: 13DPG, 1,3-diphosphoglycerate; 2PG, 2-phospho-D-glycerate; 3PG, 3-phospho-D-glycerate; 3PHP, 3-phospho-hydroxypyruvate; DHAP, dihydroxyacetone phosphate; F6P, D-fructose-6-phosphate; G1P, D-glucose-1-phosphate; G6P, D-glucose-6-phosphate; G3P, D-glyceraldehyde-3-phosphate; R5P, D-ribose-5-phosphate; RU5P, ribulose-5-phosphate; X5P, xylulose 5-phosphate.

b Negative logarithm (-Log) of *E. coli* metabolite concentrations (mol/L) predicted by SVR model.

c Negative logarithm (-Log) of *E. coli* metabolite concentrations (mol/L) predicted by NET method [16].

d Negative logarithm (-Log) of *E. coli* metabolite concentrations (mol/L) determined by prior experiments [16].

e Not available, because the metabolite is not involved in the metabolic network of *E. coli*.

f Mean of concentrations for α- and β-G1P.

g Mean of concentrations for α- and β-G6P.

h Mean of concentrations for D- and L-RU5P.

i Mean of concentrations for D- and L-X5P.

j Not determined.

In summary, the present analysis indicates that the organization of metabolic networks has a chemical basis. That is, metabolic hubs prefer to select relatively strong-polar metabolites. This basis can be explained in terms of high concentrations required by metabolic hubs to drive a variety of reactions. The present finding not only provides a molecular-level explanation to the preferential attachment principle for metabolic network expansion but also has direct implications for metabolic network design and metabolite concentration prediction.

## Materials and Methods

### Metabolic network reconstruction and topological parameter calculation

The KEGG-based metabolic network was reconstructed by manually screening the 8100 small-molecule reactions recorded in KEGG Ligand Database (http://www.genome.jp/kegg/ligand.html) (up to Sep 2009) [18]. The screening criteria are as follows: i) The reactions involving macromolecules (*e.g.*, polymers, proteins and nucleic acids) and metabolites with unspecified residues (denoted by R group) were deleted; ii) Currency metabolites, including gases, metal ions and cofactors were discarded, except that they directly participate in metabolic reactions [19], [20]. The resulting small-molecule metabolic network consists of 4875 nodes (compounds) and 9263 undirectional edges (substrate-product relations).

The metabolic network of *E. coli* was reconstructed by manually screening the 1317 small-molecule reactions for *E. coli* K-12 recorded in EcoCyc Database (http://www.ecocyc.org) [21]. The screening criteria are the same as above described. The resulting small-molecule metabolic network consists of 601 nodes (compounds) and 1538 undirectional edges (substrate-product relations).

The metabolic network of *S. cerevisiae* was reconstructed by manually screening the 1923 small-molecule reactions recorded in YEASTNET (http://www.comp-sys-bio.org/yeastnet) [22]. The screening criteria are the same as above described. The resulting small-molecule metabolic network consists of 612 nodes (compounds) and 2654 undirectional edges (substrate-product relations).

The parameters describing the network topology were calculated by Network Analyzer Plugin in Cytoscape-2.7.0 [23], [24]. The node degree of a node *n* is defined as the number of edges linked to *n*. The basic information for KEGG, *E. coli* and *S. cerevisiae* metabolites that are involved in the metabolic networks are presented in Tables S1-S5.

### Identification of early and late members of *S. cerevisiae* metabolome

To elucidate the molecular basis of preferential attachment principle underlying the evolution of metabolic networks, we identified the early and late members from *S. cerevisiae* metabolome. Recently, Prachumwat and Li classified yeast proteins into five age groups, according to the occurring patterns of their orthologs in other species [25]. The oldest age group, consisting of 1806 members, includes proteins that can be traced back to eubacterial genomes. Among these proteins, 972 are enzymes. According to the KEGG records, 633 metabolites associated with these ancient enzymes were collected, 12 of which are aerobic metabolites (according to the aerobic metabolite information provided by Raymond and Segrè [26]) and thus are not early metabolites. The remained 621 metabolites constitute the set of early metabolites of *S. cerevisiae*, in which 243 members are involved in the metabolic network of *S. cerevisiae*. The other 369 ( =  612−243) metabolites of *S. cerevisiae* metabolic network were thus regarded as late members.

### Chemical property calculation, network expansion simulation and statistical analysis

83 commonly used property descriptors were calculated with Cerius2 (Version 4.11L. Accelrys Inc. San Diego, CA.), Sybyl (Version 7.0. Tripos Associates Inc. St. Louis, MO.), Pipeline Pilot (Student Edition. Version 6.1.5. SciTegic Accelrys Inc. San Diego, CA.) and Tripos Benchware DataMiner (Version 1.6. Tripos Associates Inc. St. Louis, MO.). Stepwise multiple linear regression analysis was performed by Cerius2 (Version 4.11L. Accelrys Inc. San Diego, CA.). The numerical simulations of metabolic network expansion were performed based on python package "networkx" (version 1.2). All of the statistical analyses were performed with SPSS (Version 15.0. SPSS Inc. Chicago, IL.).

### Support vector regression model construction

By a trial-and-deletion procedure, 11 properties that have largest contributions to the support vector regression (SVR) model were selected, which include degree and 10 chemical properties, *i.e.*, 6mem rings Molecules (number of 6 membered rings), Amide Molecules (number of amide), ALogP (the Ghose and Crippen octanol-water partition coefficient), ClogP (partition coefficient octanol/water), FNSA3 (ratio of atomic charge weighted partial negative surface area on total molecular surface area), FPSA3 (ratio of atomic charge weighted partial positive surface area on total molecular surface area), HBD Count (number of hydrogen bond donating groups in the molecule), N Count (number of Nitrogen atoms), LScore Molecules (floating point Lipinski measure) and RPCG (ratio of most positive charge on sum total positive charge (Relative positive charge)). Radial basis kernel function 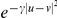 was chosen to construct a *ε*-SVR model. The parameters were trained by using grid search over supplied parameter ranges and the best parameters were obtained as follows: gamma  = 0.01, epsilon  = 0.22, cost  = 7.9. The SVR algorithm for metabolite concentration prediction is available on request.

## Supporting Information

Figure S1 Power-law degree distribution of KEGG metabolites.
(DOC)

Table S1 Basic information for KEGG metabolites that are involved in the metabolic network.
(XLS)

Table S2 Basic information for *E. coli* metabolites that are involved in the metabolic network.
(XLS)

Table S3 Basic information for *S. cerevisiae* metabolites that are involved in the metabolic network.
(XLS)

Table S4 Basic information for 80 *E. coli* metabolites that have absolute concentration values and are involved in the metabolic network.
(XLS)

Table S5 Chemical properties for hubs of KEGG-based network and organic chemical network.
(XLS)

Table S6 Absolute concentrations for *E. coli* metabolites predicted by SVR model.
(XLS)
