# Supplementary material for: Chemical Basis of Metabolic Network Organization
Source: PLoS Comput Biol. 2011 Oct 13;7(10):e1002214. doi: 10.1371/journal.pcbi.1002214 (PMC3192814; doi:10.1371/journal.pcbi.1002214)
Supplement: Figure S1 — Power-law degree distribution of KEGG metabolites. (DOC) [file pcbi.1002214.s001.doc]

**Figure S1.** Power-law degree distribution of KEGG metabolites. The number of metabolites (*N*) decays with the increase of degrees (*D*) and follows the equation *N* = *aD^-b^*.
